# The Week's Work

**Published:** 1911-08-19

**Authors:** 


### THE WEEK'S WORK.

### AN ORIGINAL HOSPITAL DESIGN.

We draw the attention of our readers to the construction of the new hospital, which has just been opened at Leicester for those who can afford only very moderate fees, and which is described in our Institutional Notes of this week. It is interesting alike to architects and to hospital officers. The architect will note, among other things, the placing of the mortuary in the basement, a position the merits or demerits of which will probably excite some diverse expressions of opinion. The hospital worker will note, probably with less mixed feelings, that the ten-bed arrangement has been adopted in the wards. The firm of architects which has been entrusted with the work is that of Messrs. Stockdale, Harrison, and Son, whose deposition would make interesting reading. How many other cases are known in which a modern mortuary forms part of the main block of hospital buildings? A census would be interesting on this point. The new hospital is also interesting to hospital managers in that it aims expressly at accommodating the very class for which the voluntary hospitals were created to cater—that class, namely, which cannot afford the fees of a nursing home and which at the same time does not care to receive free or charitable treatment. No doubt there is room for the new institution, no doubt the voluntary hospitals are overstocked with non-paying patients, no doubt also objections can be raised against the installation of pay wards. We welcome the new hospital as original in design and intention, and shall always be glad to hear of its future progress.

### THE LATE CHIEF RABBI AND THE HOSPITALS.

The late Dr. Adler, whose will has now appeared in the newspapers, has left £20 to the London Hospital, the only hospital in this country honoured similarly by him, though the King's Fund, the Metropolitan Sunday Fund, and the Zadek Hospital in Jerusalem received equivalent shares. Dr. Adler did not leave, if the reports are correct, anything at all to the so-called Jewish Hospital Memorial scheme. We do not want to waste time kicking a horse so dead as that inept proposal. But if any Jews have not already decided against it, the action of the Chief Rabbi in carefully avoiding mention of it in his will must prove conclusive. A strong man's silences are often strangely significant.

### SUPERANNUATION ALLOWANCES FOR MEDICAL OFFICERS OF HEALTH.

The object of a Bill which has been introduced in the House of Commons by Dr. Hillier is to enable medical officers of health, by definite contributions from their salaries, to provide themselves with superannuation allowances as a provision for old age and in case of permanent infirmity. The percentage amounts to be deducted annually from the salaries is from 2 per cent. to 3 per cent., according to the number of years of service at the time of the passing of the measure. The Bill makes provision for an allowance equal to fifteen-sixtieths of the average amount of the last five years' salary for an officer who has served ten years, with the addition of one-sixtieth of such average amount for every additional completed year of service until the completion of a period of service for thirty-five years, when a maximum allowance of forty-sixtieths is to be granted. The Bill has been put down for Second Reading this week, but owing to the congestion of business it is highly improbable that it will be reached. Statutory provision of a similar nature has been already made for Poor Law medical officers by the Poor Law Officers' Superannuation Act, 1896, and for the officers of asylums by the Asylums Officers' Superannuation Act, 1909.

### "HOSPITAL" IN THE "ENCYCLOPÆDIA BRITANNICA."

On pages 791-801 of the new edition of the "Encyclopædia Britannica" appears an interesting article devoted to the term "Hospital." The subject is reviewed from an entirely modern standpoint, and there is a marked difference in the treatment between the articles in the old edition and this up-to-date exposition of the essentials of the question of the status of hospitals and charitable institutions. The writer upholds the voluntary system, but pays due regard to the importance and excellence of infirmary work and Poor-law institutions. To those interested in the question the subsections "The Evolution of the Modern Hospital" and "The Hospital City" will appeal especially, since they are devoted to a discussion of two very important matters. The writer points out that the large modern hospitals are in many ways independent communities with many hundreds of individuals. This is especially the case in those English, Continental, and American institutions which are built on modifications of the pavilion system. The writer lays great, and, in our opinion, well-deserved, stress on the importance of further extending this ideal of independence—or, rather, self-dependence—by evolving the hospital city; and the sketch he furnishes of such a city in working demands careful consideration. The problem of hospital administration is fully dealt with, and a résumé is given of the arguments in favour of paying wards and contributions towards hospitals that are now wholly supported by voluntary contributions. Finally, the important subject of hospital construction is discussed, and we note that stress is laid on the advisability of providing a separate and independent admission block or department—a matter which is so often treated with scant attention by hospital architects. A model plan of such an admission block is given, and the whole sub-section dealing with construction is clearly and concisely written, prominence being given to those matters which demand more than passing consideration. We commend the whole article, which is one of the best in the new edition, to the attention of hospital workers, since it is a fair statement of the case for the voluntary principle, in which the case for the other side has not been neglected. A short bibliography is given at the end. Doubtless considerations of space prevented a more extended review of hospital literature, but it is somewhat to be regretted that this bibliography has not been made fuller. For instance, Merke and Ruppel's elaborate monograph in Weyl's Handbuch d. Hygiene is not mentioned, nor the equally elaborate report of the Belgian Commission on hospital construction.

### THE NEW CHAIRMAN OF THE SWANSEA HOSPITAL.

Since Mr. Roger Beck has been unable to see his way again to accept the chairmanship of the Swansea Hospital this year, the Board of the institution has approached Mr. F. W. Gibbins, of Garthmor, Neath, who has accepted the honour. Mr. Gibbins was High Sheriff of the county of Glamorgan during the past year, and is manager of the Eagle Tinplate Company, Neath. While sharing the Board's regret at Mr. Beck's decision, we are very glad to welcome his successor in the chairmanship, whose position in the industrial world no doubt will engage the continual and increasing support of the working classes.

### A NOTEWORTHY OMISSION.

The hospital world cannot help expressing its regret that the will of the late Dame Charlotte Russell, which will benefit so many charitable institutions, apparently contains no single bequest to hospitals. Lady Russell, relict of Sir Nichol Russell, Kt., was the daughter of Dr. Lorimer, M.D., a man well known in his day as Deputy Inspector of Hospitals in Madras, and it is curious to note, where so many branches of charity received benefactions from her large estate, that that branch to which her father devoted most of his life should be unrepresented among them.

### DENTISTRY AND ANÆSTHETICS AT POPLAR.

At a meeting of the London County Council on August 1, the Education Committee reported that the scheme sanctioned by the Council for the dental treatment, at the Poplar Hospital for Accidents, of children attending public elementary schools, had been in operation since March. The governing body of the hospital now stated that the dentist regarded the use of anæsthetics as indispensable in difficult cases of extraction, and suggested that the senior resident surgeon of the hospital should be appointed to administer anæsthetics. It was proposed that cases requiring anæsthetics should be dealt with on one afternoon a fortnight and that the anæsthetist should be paid at the rate of £25 a year. As a general rule, the Committee was of opinion that provision should not be made for the treatment, at centres established by committees of medical practitioners, of cases requiring the use of anæsthetics. In the case of the Poplar Hospital, however, the anæsthetics would be administered by a surgeon on the hospital staff, and they thought that in this instance the request of the governing body of the hospital should be acceded to. The surgeon had already dealt with the difficult cases which had arisen, and it would be necessary to make payment to the hospital for these cases. The Council agreed to adopt the suggestion of the governing body of the hospital, and we think that its wisdom in doing so is hardly open to dispute.

### THE WELSH MEMORIAL TO KING EDWARD.

As everyone now knows, the memorial of Wales to the late King is taking the form of a propagandist and general campaign against consumption. We understand that nearly £190,000 has been promised for this purpose, and that during the winter Welsh villages will be traversed by caravans. It will be remembered that perhaps the most striking part of the campaign has been its association with the reconstruction of Cardiff Infirmary, which will be rechristened after King Edward when the necessary funds have been subscribed in full.

### HOSPITAL BEQUESTS FROM A FAMOUS AUTHOR.

The late Sir W. S. Gilbert, author of the "Bab Ballads" and "The Mikado," who left approximately £112,000 gross estate, has bequeathed the bronze statuette of himself by Andrea Lucchesi to the Matron, Miss Cara E. Pillans, of the Cottage Hospital on Bushey Heath. He also expressed a wish that Lady Gilbert should continue his annual subscription of twenty guineas to the "Kitty Cot" in the Victoria Hospital for Children at Chelsea, and that the Bushey Cottage Hospital should continue to receive ten guineas annually from his estate. It is interesting to remember that Sir Arthur Sullivan, who died in 1900, left estate valued at £54,527 gross.

### AN URGENT NEED AT ST. MARY'S HOSPITAL.

Anyone who has not visited S. Mary's Hospital, and needs convincing, which he would not need if he went there, that its casualty department requires remodelling at once, is referred to the expert statement of the case which we were glad to publish in The Hospital of May 6 (page 143). We mention the subject again as the Coronation somewhat interfered with the progress of its appeal for £7,500 for this purpose. All that facts and argument could do we have done already. Now we boldly appeal to the sentimental propensities of the richer public, in the holiday leisure of which charitable feelings surely may claim a genuine share.

### THE SHROPSHIRE CAMPAIGN AGAINST CONSUMPTION.

The King Edward Memorial in Shropshire, which has taken the form of a sanatorium of thirty beds at Shirlett, near Much Wenlock, has begun its practical public functions by its opening at the hands of Princess Alexander of Teck. The county of Shropshire possesses an Association for the Prevention of Consumption, of which Lord Kenyon is chairman, and he assured his hearers that of the £10,500 which the new buildings had cost £9,500 have been subscribed. In what light does the Association regard the Government's sanatoria proposals; is it, like the joint hospital boards, as we noted in our issue of July 29 (page 441), hoping to secure a share of the special building fund? It would be very well advised to make clear its attitude on this question, as its support from the public and their confidence in the Association will depend very much on its answers to these questions. Sanatoria supported voluntarily have shown to the Insurance Bill a culpable indifference and maintained a silence which is not likely to increase the confidence of their well-wishers. The National Sanatorium now at Bournemouth is a notable exception to this bad rule.

### HOSPITAL CONCERTS IN GERMANY.

It is announced that an orchestral society of medical men has been founded in Berlin, and the products of its concerts are to be devoted to medical benevolent funds. One is naturally tempted to ask whether that somewhat vague term is intended to include the funds of the hospitals to the staffs of which the medical musicians belong. Germany, the country of music, is however only following the example of Paris, where we understand a similar society exists. The possibility of a medical orchestra for the benefit of London hospitals is, we fear, remote. Our lethargic adoption of new practices, however, has the advantage of enabling us to profit by the experience of foreigners to a degree that has sometimes put us ahead of them in the long run. In the present instance it is perhaps to be doubted whether there is either sufficient love of music or sufficient talent to maintain an institutional orchestra in the Metropolis on commercial lines. Nevertheless a glance at the article which we published last week on "Twenty-three Years at Bethlem" will show what an energetic medical superintendent was able to do in encouraging orchestral material among mental patients, and perhaps the funds of the voluntary hospitals in London will yet be recruited from this apparently more promising source. The same objections as were raised recently to Sunday cinematograph shows could hardly apply to Sunday medical concerts.

### SPRING CLEANING THE WARDS IN AUGUST.

The annual turn-out which is performed in private houses somewhere about April, or even earlier, has to be postponed in institutions to a less busy time of year; though why indeed August, which is chosen by the institutions, should not be chosen also by middle-class families when their absence on holidays would obviate the necessary inconvenience, is a mystery that some householder, who is also a hospital officer, must be left to explain. While we write the cleaning process is busy in the hospitals of London. From Monday to Thursday the out-patient department of the Westminster Hospital was closed—the Coronation stand allowed the wards to be cleaned in June as was stated in a note beneath the photograph published in The Hospital of July 1, page 343. The out-patient department at the Royal Free Hospital will remain closed until September 4, though urgent cases will be received at the main entrance. Until the 31st the Western Ophthalmic Hospital will be closed entirely to patients, and, as The Hospital announced on June 3, page 253—the actual day of closing—the Paddington Green Hospital for Children is closed until October 1, when its new out-patient unit will be opened. The rank-and-file of the domestic staff in the voluntary hospitals are having a busy time as usual this holiday season.

### "THE WILL IN THE HAT CASE."

The Harris Norman bequest, often known as the will in the hat case, which has been in litigation for the past three years, has now been settled, and the Governors of Addenbrooke's Hospital have received as their moiety of the estate, after payment of litigation, £4,972. It will be remembered that Harris Norman, who had received treatment at Addenbrooke's Hospital in his lifetime, when in a very impecunious state, bequeathed half of his estate of between £11,000 and £12,000 to Addenbrooke's Hospital and the other half to the "London Jewish Synagogue for poor and needy Jews," and appointed "The Governor" of Addenbrooke's Hospital as sole executor. The will, which was made on half a sheet of notepaper, was found in the lining of a hat which the deceased used to wear, and which he gave to one of his few acquaintances. The litigation was accounted for by the fact that there was no such Jewish synagogue as that described by the deceased, and it had to be decided as to what institution was intended by the deceased's description. The next-of-kin (far removed) therefore made an attempt to upset the will. This reminds us curiously of the Bateman case, which was discussed in The Hospital of March 18 (page 715). There Mrs. Bateman left a share of her residuary estate to an imaginary "Royal Hospital for Women," which brought six hospitals as claimants into the Courts. As we said on that occasion, "though we should be the last people in the world to discourage legators to hospitals, generous impulses which involve half-a-dozen charities in litigation we are better without." Addenbrooke's is to be congratulated on the substantial sum which the payment of legal costs has left for its charitable uses.

### THIS WEEK'S DRUG MARKET.

The drug market has again been very quiet during the week, and in fact business is not likely to be active until autumn. The dock strike has had little effect on prices, but the delay in the delivery of goods occasioned considerable inconvenience. Price changes have been few and for the most part unimportant. The position of opium is unchanged. Chamomiles are dearer, as also is valerian. Oil of cloves is also somewhat higher in price. Very high prices are quoted for hydrastis canadensis.

